# Supplementary material for: Neuronal Dynamics and miRNA Signaling Differ between SH-SY5Y APPSwe and PSEN1 Mutant iPSC-Derived AD Models upon Modulation with miR-124 Mimic and Inhibitor
Source: Cells. 2021 Sep 14;10(9):2424. doi: 10.3390/cells10092424 (PMC8465877; doi:10.3390/cells10092424)
Supplement: Supplementary file 1 [file cells-10-02424-s001.zip › cells-1353955-SI.pdf]

SUPPLEMENTARY DATA

Neuronal dynamics and miRNA signaling differ in SH-SY5Y *APPSwe* and *PSEN1* mutant iPSC-derived AD models by miR-124 mimic and inhibitor modulation

Cells

Gonçalo Garcia [1,2], Sara Pinto [1,3], Mar Cunha [1], Adelaide Fernandes [2,4], Jari Koistinaho [5,6], and Dora Brites [1,*]

1 Neuroinflammation, Signaling and Neuroregeneration Laboratory, Research Institute for Medicines (iMed.ULisboa), Faculty of Pharmacy, Universidade de Lisboa, 1649-003 Lisboa, Portugal; ggarcia@campus.ul.pt; sfcpinto@campus.ul.pt; mariana.xcf@gmail.com; dbrites@ff.ul.pt
2 Department of Pharmaceutical Sciences and Medicines, Faculty of Pharmacy, Universidade de Lisboa, 1649-003 Lisboa, Portugal; amaf@ff.ulisboa.pt;
3 Instituto de Medicina Molecular, Faculdade de Medicina, Universidade de Lisboa, 1649-003 Lisboa, Portugal;
4 Central Nervous System, Blood and Peripheral Inflammation, Research Institute for Medicines (iMed.ULisboa), Faculty of Pharmacy, Universidade de Lisboa, 1649-003 Lisboa, Portugal
5 A.I. Virtanen Institute for Molecular Sciences, University of Eastern Finland, FI-70211 Kuopio, Finland; jari.koistinaho@uef.fi
6 Neuroscience Center, Helsinki Institute of Life Science (HiLIFE), FI-00014 University of Helsinki, Helsinki, Finland; jari.koistinaho@helsinki.fi
* Correspondence: dbrites@ff.ul.pt; Tel.: (+351 217946450)

Supplementary Tables

Table S1. Data on the healthy control and the Alzheimer's disease (AD) patient from which induced pluripotent stem cells (iPSCs) were generated and differentiated into iNeurons that we used in this study.

Alias	Cell line	Sex	Age at biopsy	Mutation genotype	*APOE* genotype	Health status	Sample origin	Reprogramming method	Karyotype	Reference
CTRL	Ctrl1	F	Adult	-	*ε3/ε3*	Healthy	Skin biopsy	SeV 1.0	46XX Normal	[32,33]
pPSEN	AD3	F	47 y	*PSEN1ΔE9 deletion*	*ε3/ε3*	Pre-symptomatic AD patient	Skin biopsy	SeV 2.0	46XX Normal	[32,33]

Apolipoprotein E (*APOE*); iPSC-derived iNeurons from Ctrl1 were designed as iNEU-*WT*, while those from AD3 were denominated iNEU-*PSEN* in the present study; F, female; y, years; SeV, Sendai virus.

Table S2. Total RNA and protein concentrations in exosomes isolated from SH-*WT*/SW-*SWE* and iNEU-*WT*/iNEU-*PSEN* neuronal cells.

	SH-SY5Y neuroblastoma cells		iPSCs-generated iNeurons	
	**SH-*WT***	**SW-*SWE***	**iNEU-*WT***	**iNEU-*PSEN***

Total RNA (ng/µL)	5.79 ± 0.43	5.67 ± 0.45	12.19 ± 0.45 **	19.69 ± 1.63 #
Total Protein (µg/µL)	0.24	0.35	1.32	1.46

SH-*WT*, human SH-SY5Y wild-type (*WT*) neurons; SH-*SWE*, human SH-SY5Y neurons transfected with the *APP695 Swedish* mutant protein; iNEU-*WT*, iNeurons differentiated from human induced pluripotent cells (iPSCs) generated from fibroblasts from a healthy control using specific protocols [32,35]; iNEU-*PSEN*, iNeurons differentiated from iPSCs of a pre-symptomatic individual carrying the *PSEN1ΔE9* mutation. Exosomes were isolated by differential ultracentrifugation as described in Materials and Methods. Total RNA (3 replicates) and protein (3 pooled samples) concentrations are expressed as mean ± SEM. ** $p < 0.01$ vs. SH-WT; # $p < 0.05$ vs. iNEU-WT; two-tailed Student's *t* test.

Table S3. - List of primers used for mRNA/miRNA analysis by RT-qPCR.

**miRNAs**	**Target sequence**
hsa-miR-124-3p	UAAGGCACGCGGUGAAUGCC
hsa-miR-125b-5p	UCCCUGAGACCCUAACUUGUGA
hsa-miR-21-5p	UAGCUUAUCAGACUGAUGUUGA
hsa-miR-146a-5p	UGAGAACUGAAUUCCAUGGGUU
hsa-miR-155-5p	UUAAUGCUAAUCGUGAUAGGGGU
UniSp6 (spike-in)	(Reference sequence)
U6	(Reference sequence)
**mRNAs**	**Primer sequence (5′ to 3′)**
APP	**FWD:** GCTGGTGGAGACACACATGGCC<br>**REV:** GGATCTGAGCGGCTTTCTTGGG
Dynein	**FWD:** GCCTCAGTCTCTGTCCCATC<br>**REV:** AAGTCCTGGGGTAAGGTGCT
KIF3A	**FWD:** TGGCAGCTAAAATGTGTTGC<br>**REV:** CTGTCTTTGGCCTTGCTTTC
GAPDH	**FWD:** CGCTCTCTGCTCCTCCTGTT<br>**REV:** CCATGGTGTCTGAGCGATGT
β-actin	**FWD:** ACAGAGCCTCGCCTTTGCCG<br>**REV:** TGGGCCTCGTCGCCCACATA

hsa, *Homo sapiens*; miR, microRNA; mRNA, messenger RNA; RT-qPCR, real-time quantitative polymerase chain reaction; FWD, forward; REV, reverse; *APP*, amyloid precursor protein coding gene; KIF3*A* kinesin-like protein coding gene; *GAPDH*, glyceraldehyde 3-phosphate dehydrogenase protein coding gene.

Table S4 – Alterations in cell area after transfection with mock, miR-124-3p inhibitor and mimic in SH-*WT* and SH-*SWE* cells.

	Cell area (μm^2)		
	**mock**	**inhibitor**	**mimic**
**SH-*WT***	1037 ± 213	1398 ± 330	961 ± 294
**SH-*SWE***	1437 ± 305	1453 ± 262	1133 ± 189

Cells were differentiated, modulated with miR-124 inhibitor and mimic, and their exosomes isolated as described in Material and Methods. Data are mean area ± SEM from at least five independent experiments. These cell sizes were used to normalize the MitoTtracker fluorescence intensity presented in Figure 3C. SH-*WT*, human SH-SY5Y wild-type neurons; SH-*SWE*, human SH-SY5Y neurons transfected with the *APP695 Swedish* mutant protein; No significant differences were found.

Table S5 – Expression profile of inflammatory-associated miRNAs assessed by RT-qPCR in SH-*WT* and iNEU-*WT* cells, as well as in their exosomes, after transfection with miR-124-3p inhibitor and mimic.

	SH-*WT* cells		iNEU-*WT* cells	
	miR-124 inhibitor	miR-124 mimic	miR-124 inhibitor	miR-124 mimic
**miR-125b**	0.39 ± 0.21 *	5.25 ± 1.14 **	0.3 ± 0.23 *	2.55 ± 2.28
**miR-21**	0.86 ± 1.04	9.35 ± 3.21 *	3.56 ± 0.83 **	1.13 ± 0.09
**miR-146a**	1.63 ± 0.49 *	0.36 ± 0.34 #	0.56 ± 0.34	0.14 ± 0.2 **
**miR-155**	4.6 ± 3.12 *	0.64 ± 0.52	0.35 ± 0.05 **	0.03 ± 0.01 **

Cells were differentiated, modulated with miR-124 inhibitor and mimic, and their exosomes isolated as described in

	SH-*WT* exosomes		iNEU-*WT* exosomes	
	miR-124 inhibitor	miR-124 mimic	miR-124 inhibitor	miR-124 mimic
**miR-125b**	0.31 ± 0.1 ##	4.33 ± 1.23 **	0.44 ± 0.05 **	2.27 ± 0.54 **
**miR-21**	0.64 ± 0.24 #	6 ± 2.28 **	0.37 ± 0.1 *	2.04 ± 0.65 *
**miR-146a**	0.61 ± 0.45	1.98 ± 0.38 #	0.59 ± 0.2 #	3 ± 0.43 **
**miR-155**	9.88 ± 1.1 **	0.22 ± 0.37 ##	3.03 ± 0.09 **	0.37 ± 0.39 ##

Material and Methods. Data are mean ± SEM fold-change from at least three independent experiments. * $p < 0.05$ and ** $p < 0.01$ vs. respective mock controls; One-way ANOVA with Bonferroni post hoc test. # $p < 0.05$ and ## $p < 0.01$ vs. respective mock controls; two-tailed Student's *t* test. SH-*WT*, human SH-SY5Y wild-type neurons; SH-*SWE*, human SH-SY5Y neurons transfected with the *APP695 Swedish* mutant protein; iNEU-*WT*, iNeurons differentiated from human induced pluripotent cells (iPSCs) generated from fibroblasts from a healthy control using specific protocols [32,35]; iNEU-*PSEN*, iNeurons differentiated from iPSCs of a pre-symptomatic individual carrying the *PSEN1ΔE9* mutation.

Supplementary Figures

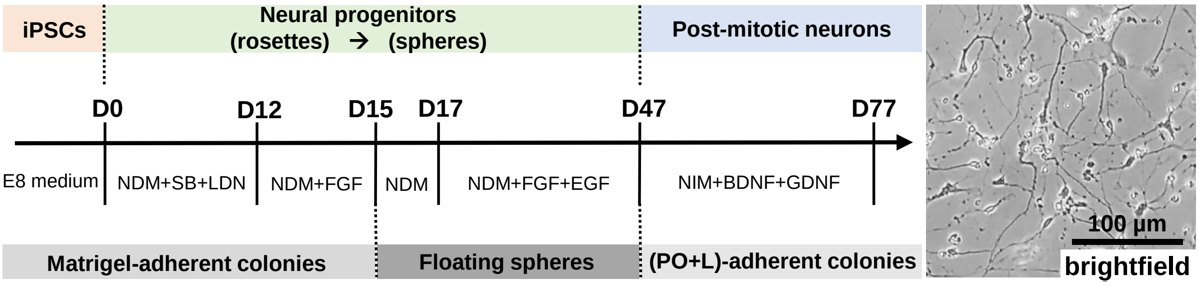

Figure S1. Schematic representation for the differentiation of induced Pluripotent Stem Cells (iPSCs) into induced neurons (iNeurons), following specific protocols [32,35], as described in Materials and Methods. Essential 8™ medium (E8 medium; Thermo Fischer Scientific) was firstly used for growth and expansion of iPSCs after plating on Matrigel-coated plates, as described in Material and Methods section. Differentiation was initiated by replacing the medium to neural differentiation medium (NDM) containing SB431542 (SB) and LDN193189 (LDN) until day 12. Then, the medium was changed to NDM containing fibroblast growth factor (FGF) until day 15 to expand rosettes, and to NDM alone until day 17, to allow neural progenitor sphere formation. After that, we used NDM with FGF and epidermal growth factor (EGF) until day 47. Cells were platted in Poly-L-Ornithine + Laminin (PO+L) and differentiated with neuron induction medium (NIM) containing brain-derived neurotrophic factor (BDNF) and glial cell line-derived neurotrophic factor (GDNF) until D77. The process was monitored using brightfield microscopy, as represented by the image obtained prior to experiments.

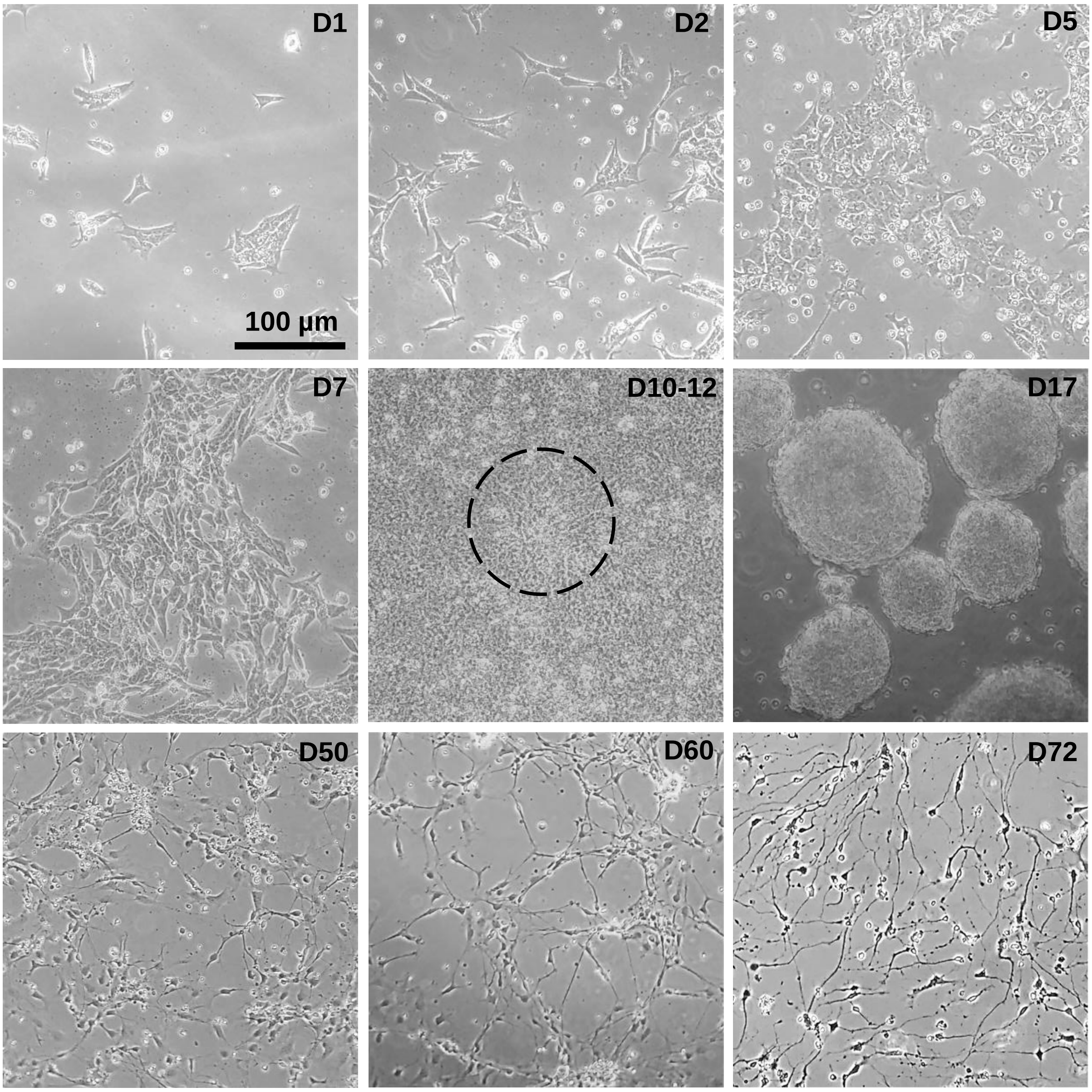

Figure S2. Representative brightfield images of the sequential differentiation steps according to the schematic protocol in the Supplementary Figure S1, and as described in Material and Methods. First, we originate the growth and expansion of induced pluripotent stem cells (D1, day one; D2, day two; D5, day five; and D7, day seven), followed by the sequential formation and expansion of rosettes (dashed circle) from D10 to D12, before cell detachment to form neurospheres (D12). Neurospheres were maintained and expanded until D47, when they were dissociated, plated into Poly-L-Ornithine + Laminin-coated plates and matured with brain-derived neurotrophic factor (BDNF) and glial cell line-derived neurotrophic factor (GDNF) until D77.

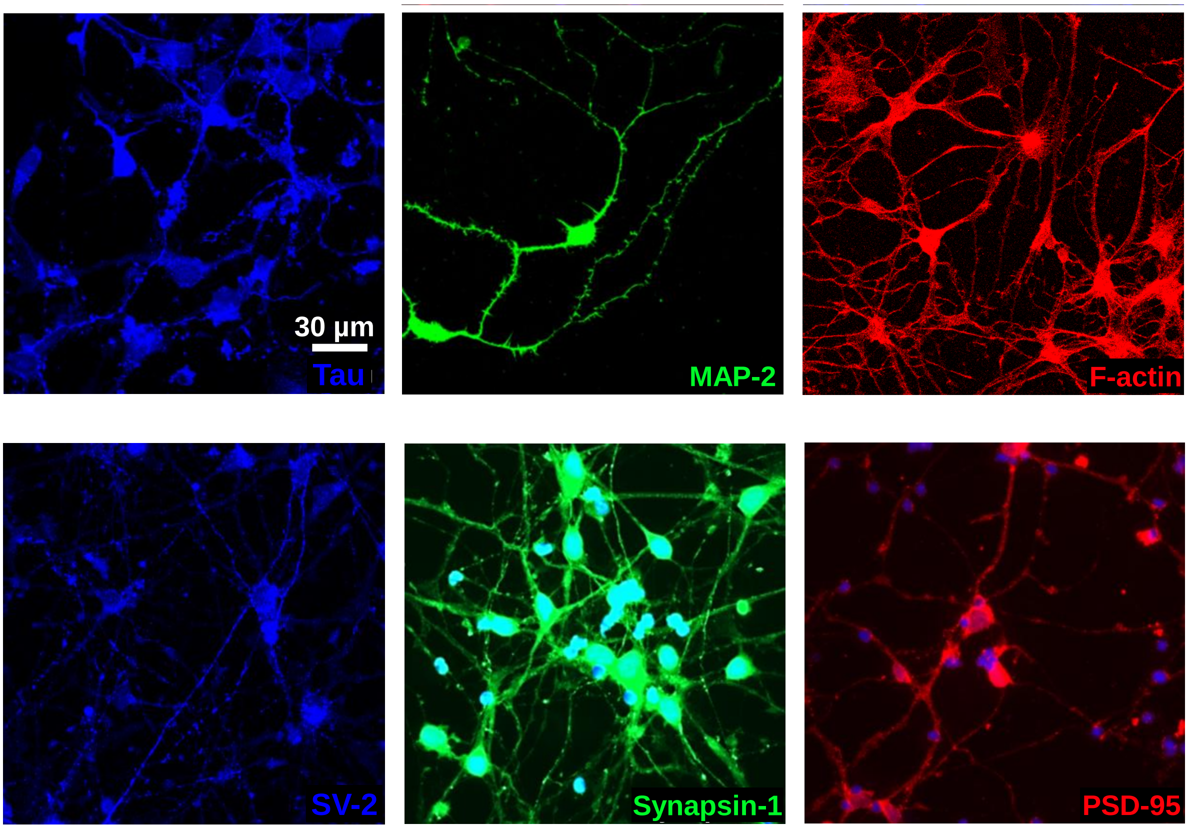

Figure S3. Representative fluorescence microscopy images of cytoskeletal and synaptic proteins in induced neurons (iNeurons) differentiated from Induced Pluripotent Stem Cells (iPSCs) and maturated following specific protocols [32,35], as described in Materials and Methods. After day 77, neuronal cells display a branching morphology (Supplementary Figure S1, brightfield image) and express the microtubule-associated proteins Tau and MAP-2, as well as the cytoskeleton-associated F-actin patterns in dendrites and axons. Expression of the pre-synaptic markers, synaptic vesicle-2 (SV-2) and synapsin-1, together with the post-synaptic density-95 (PSD-95) marker, are in conformity with iNeuron synaptic maturation.

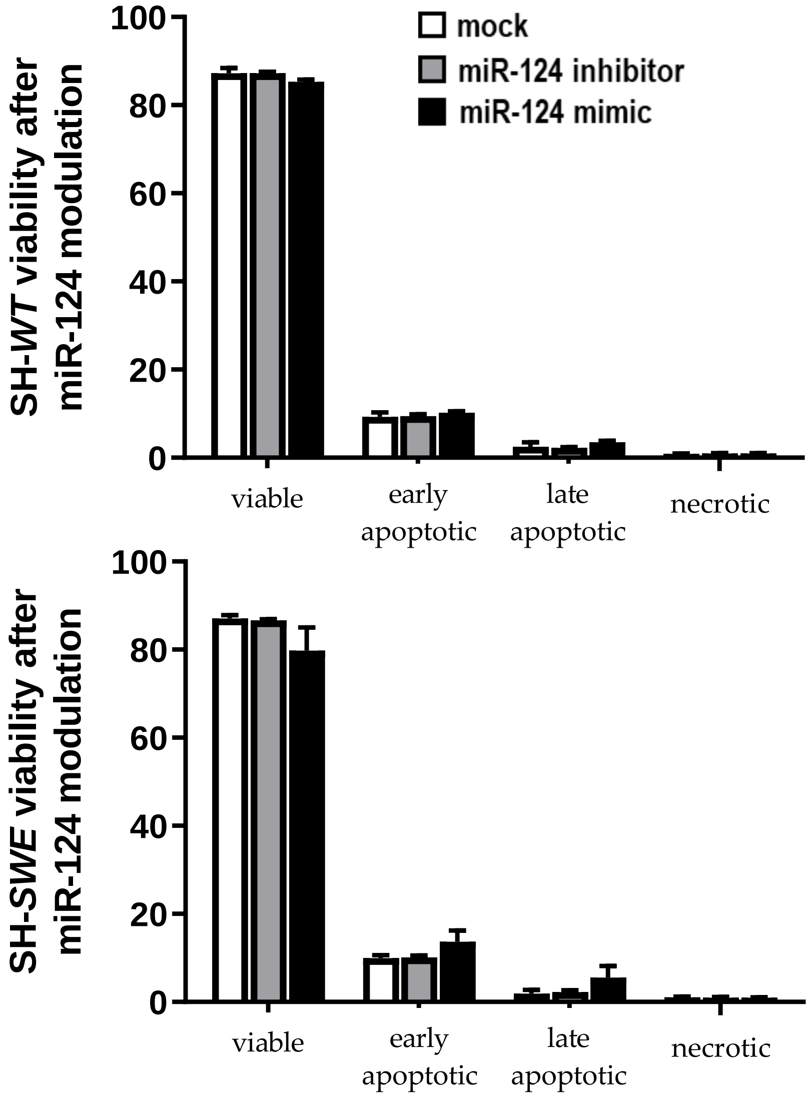

Figure S4. Modulation of miR-124 with inhibitor (anti-miR-124-3p) and mimic (pre-miR-124-3p) does not influence viability of SH-*WT* and SH-*SWE* cells, relatively to transfected mock control. Cell viability was assessed as described in Materials and Methods by flow cytometry analysis. Four cell populations were distinguished: viable (V-PE and 7-AAD double-negative), early apoptotic (V-PE positive and 7-AAD negative), late apoptotic (V-PE and 7-AAD double-positive), and necrotic cells/cellular debris (V-PE negative and 7-AAD positive). Data are shown as the mean percentage (± SEM), from at least three independent experiments. SH-*WT*, human SH-SY5Y *w*ild-type neurons; SH-*SWE*, human SH-SY5Y neurons transfected with the *APP(695) Swedish* mutant protein.

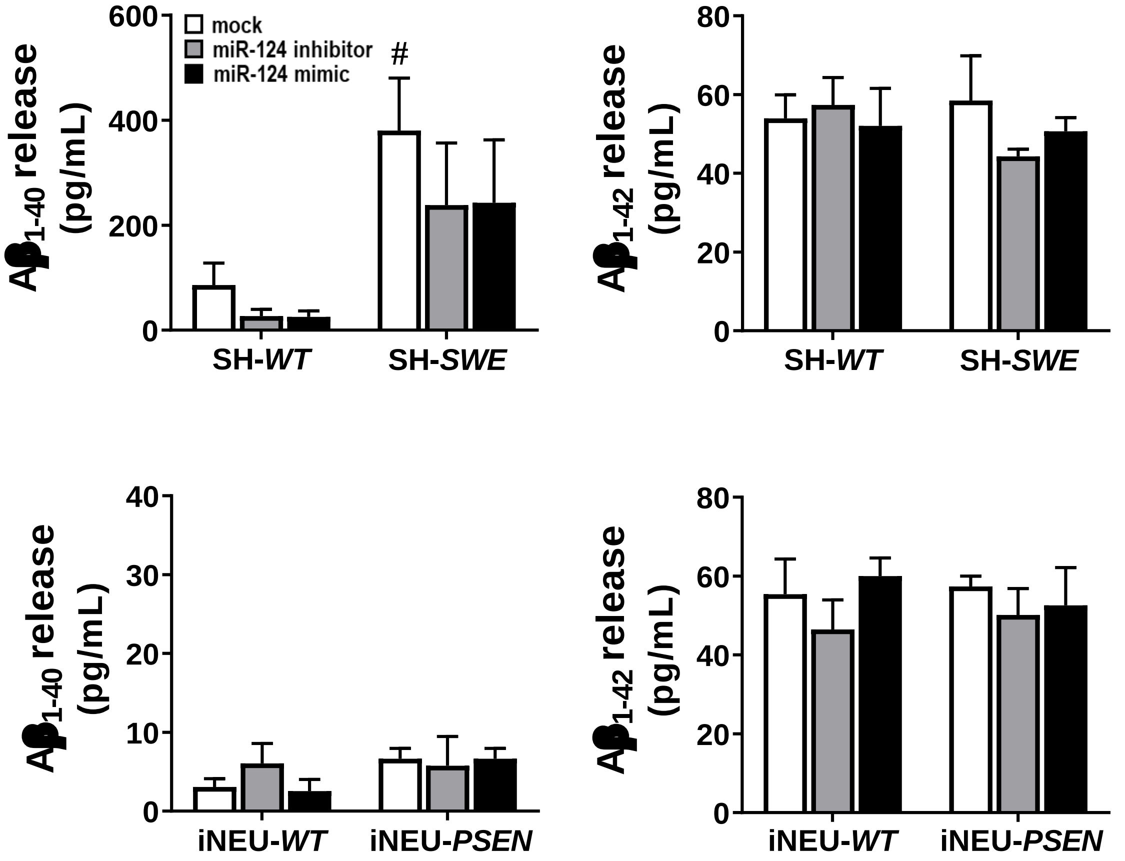

Figure S5. Levels of amyloid-beta peptide (Aβ)1-40 and Aβ1-42 in the secretome of differentiated SH-*WT*/*SWE* and iNEU-*WT*/*PSEN* cells, before and after miR-124 modulation with inhibitor and mimic, as described in Materials and Methods. Results of at least three independent experiments are shown as mean ± SEM fold-change vs. SH-*WT* cells. $^{\#}$ $p < 0.05$ (two-tailed Student's *t* test). SH-*WT*, human SH-SY5Y *w*ild-type neurons; SH-*SWE*, human SH-SY5Y neurons transfected with the *APP695 Swedish* mutant protein; iNEU-*WT*, iNeurons differentiated from human induced pluripotent cells (iPSCs) generated from fibroblasts from a healthy control using specific protocols [*32*,*35*]; iNEU-*PSEN*, iNeurons differentiated from iPSCs of a pre-symptomatic individual carrying the *PSEN1ΔE9* mutation.
